# Trans-ethnic meta-analysis of genome-wide association studies identifies maternal *ITPR1* as a novel locus influencing fetal growth during sensitive periods in pregnancy

**DOI:** 10.1371/journal.pgen.1008747

**Published:** 2020-05-14

**Authors:** Fasil Tekola-Ayele, Cuilin Zhang, Jing Wu, Katherine L. Grantz, Mohammad L. Rahman, Deepika Shrestha, Marion Ouidir, Tsegaselassie Workalemahu, Michael Y. Tsai

**Affiliations:** 1 Epidemiology Branch, Division of Intramural Population Health Research, *Eunice Kennedy Shriver* National Institute of Child Health and Human Development, National Institutes of Health, Bethesda, Maryland, United States of America; 2 Division of Intramural Population Health Research, *Eunice Kennedy Shriver* National Institute of Child Health and Human Development, National Institutes of Health, Bethesda, Maryland, United States of America; 3 Department of Population Medicine and Harvard Pilgrim Healthcare Institute, Harvard Medical School, Boston, Massachusetts, United States of America; 4 Department of Laboratory Medicine and Pathology, University of Minnesota, Minneapolis, Minnesota, United States of America; Cincinnati Children's Hospital Medical Center, UNITED STATES

## Abstract

Abnormal fetal growth is a risk factor for infant morbidity and mortality and is associated with cardiometabolic diseases in adults. Genetic influences on fetal growth can vary at different gestation times, but genome-wide association studies have been limited to birthweight. We performed *trans*-ethnic genome-wide meta-analyses and fine mapping to identify maternal genetic loci associated with fetal weight estimates obtained from ultrasound measures taken during pregnancy. Data included 1,849 pregnant women from four race/ethnic groups recruited through the NICHD Fetal Growth Studies. We identified a novel genome-wide significant association of rs746039 [G] (*ITPR1*) with reduced fetal weight from 24 to 33 weeks gestation (*P*<5x10^-8^; log_10_BF>6). Additional tests revealed that the SNP was associated with head circumference (*P* = 4.85x10^-8^), but not with abdominal circumference or humerus/femur lengths. Conditional analysis in an independent sample of mother-offspring pairs replicated the findings and showed that the effect was more likely maternal but not fetal. Trans-ethnic approaches successfully narrowed down the haplotype block that contained the 99% credible set of SNPs associated with head circumference. We further demonstrated that decreased placental expression of *ITPR1* was correlated with increased placental epigenetic age acceleration, a risk factor for reduced fetal growth, among male fetuses (r = -0.4, *P* = 0.01). Finally, genetic risk score composed of known maternal SNPs implicated in birthweight among Europeans was associated with fetal weight from mid-gestation onwards among Whites only. The present study sheds new light on the role of common maternal genetic variants in the inositol receptor signaling pathway on fetal growth from late second trimester to early third trimester.

**Clinical Trial Registration:** ClinicalTrials.gov, NCT00912132.

## Author summary

Abnormal fetal growth is a risk factor for infant morbidity and mortality, and adult cardiometabolic diseases. Genetic influences on fetal growth can vary at different gestation times. We performed *trans*-ethnic genome-wide meta-analyses of 1,849 pregnant women from four race/ethnic groups to identify maternal genetic loci associated with ultrasound-based fetal weight estimates at three gestational periods. We identified and validated a novel genome-wide significant association of rs746039 [G] in the *ITPR1* gene with reduced fetal weight at end of second trimester. We further demonstrated that decreased placental expression of *ITPR1* was correlated with increased placental epigenetic age acceleration, a risk factor for reduced fetal growth, among male fetuses. We evaluated known birthweight loci and identified gestation time-specific associations of six maternal loci with fetal weight. A maternal genetic risk score of birthweight was associated with fetal weight from mid-gestation onwards among Whites. Our study sheds new light on the genetic regulation of gestation time-specific fetal growth.

## Introduction

Fetal growth is an important predictor of neonatal morbidity and mortality [1], childhood morbidity [2, 3], and adulthood risk of cardiometabolic diseases including type 2 diabetes, obesity, cardiovascular diseases and cognitive dysfunction [4–6]. Maternal genetic variations and environmental factors, and placental function are important determinants of fetal growth [7, 8]. It is increasingly understood that fetal growth trajectories are nonlinear, and the growth patterns vary by gestation time [9, 10]. Offspring birthweight alone cannot represent the pattern of intrauterine growth at different times during gestation [11]. Previous studies have demonstrated that the genetic contribution to fetal growth, relative to *in-utero* environmental influences, varies over gestation [11–13]. Therefore, different genetic loci may influence fetal growth at different times of gestation, and the same genetic locus may have different effects on fetal growth due to its varied interactions with the *in-utero* environment by gestation timing [14].

To date, genetic studies of maternal genetic influences on fetal growth at different windows of gestation are lacking. Previous genome-wide association studies (GWASs) on fetal growth have been mainly focused on birthweight. These studies, performed in predominantly European ancestry populations, have discovered 209 single nucleotide polymorphisms (SNPs) in the maternal and fetal genome associated with offspring birthweight [15–19]. Using a method that partitions SNP heritability into maternal and fetal components [20], 7.6% and 28.5% of the variance in birthweight was attributed to maternal and fetal SNPs on the genotyping arrays, respectively [19]. Maternal genetic contributions to offspring birthweight can be due to maternally inherited offspring risk alleles or the influence of maternal genotypes on components of the intrauterine environment [18, 21]. Identifying maternal genetic loci that influence fetal growth by modulating the intra-uterine environment has a potential to elucidate *in-utero* mechanisms that underlie aberrant fetal growth and its links with future risk of adult cardio-metabolic diseases [22].

In the present study, we performed the first *trans*-ethnic maternal GWAS of fetal weight among pregnant women from four self-identified race/ethnic groups in the U.S. (Whites (n = 580), Blacks (n = 556), Hispanics (n = 508), and East Asians (n = 205)) with high quality longitudinal fetal sonography data. Specifically, we performed *trans*-ethnic GWAS meta-analyses and fine mapping to identify maternal genetic loci associated with fetal weight at different gestation times during pregnancy (i.e. at the end of first (13 weeks and 6 days of gestation), second (27 weeks and 6 days), and third trimester of pregnancy (40 weeks and 0 days)). An independently genotyped data set of mother-offspring dyads was used to validate the association and to differentiate whether the effect was more likely due to direct maternal or fetal genetic influence. To understand the potential *in-utero* mechanism linking the maternal genetic locus with fetal growth, we evaluated whether decreased expression of the implicated gene was associated with accelerated epigenetic aging of the placenta, an early tissue senescence associated with increased risk of low birthweight [23, 24]. Finally, we evaluated whether previously known maternal GWAS loci associated with birthweight also influence fetal weight across 13–40 weeks gestation. We also tested associations between genetic risk score (GRS) generated using the maternal GWAS loci and fetal weight during gestation. Our approach that integrated *trans*-ethnic GWAS in longitudinal fetal biometric data identified a common variant in the inositol 1,4,5-triphosphate receptor type 1 gene associated with gestation-specific fetal weight and follow-up characterization of the placental gene expression and DNA methylation-based aging suggested that its effect on fetal weight may be mediated through accelerated aging of the placenta.

## Results

### Data set

The characteristics of our study participants by race/ethnicity groups are presented in S1 Table. More details about the study participants has been described previously [10]. The mean ± standard deviation (s.d.) maternal age was 30.3±4.5, 25.4±5.3, 27.1±5.5, and 30.8±4.6 years for Whites, Blacks, Hispanics, and East Asians, respectively. The mean ± s.d. gestational age at delivery was 39.5±1.1 weeks for each race/ethnicity group. Fetal weights at 13, 27, and 40 weeks gestation were highest among Whites and lowest among Asians. Maternal age, pre-pregnancy body mass index and parity had weak positive correlations with fetal growth measures (S2 Table). These maternal characteristics were not adjusted for in the genome-wide models because they may be in the pathway linking the genetic loci with fetal growth measures.

### Novel association of an *ITPR1* locus with fetal weight and head circumference

**Fig 1:**
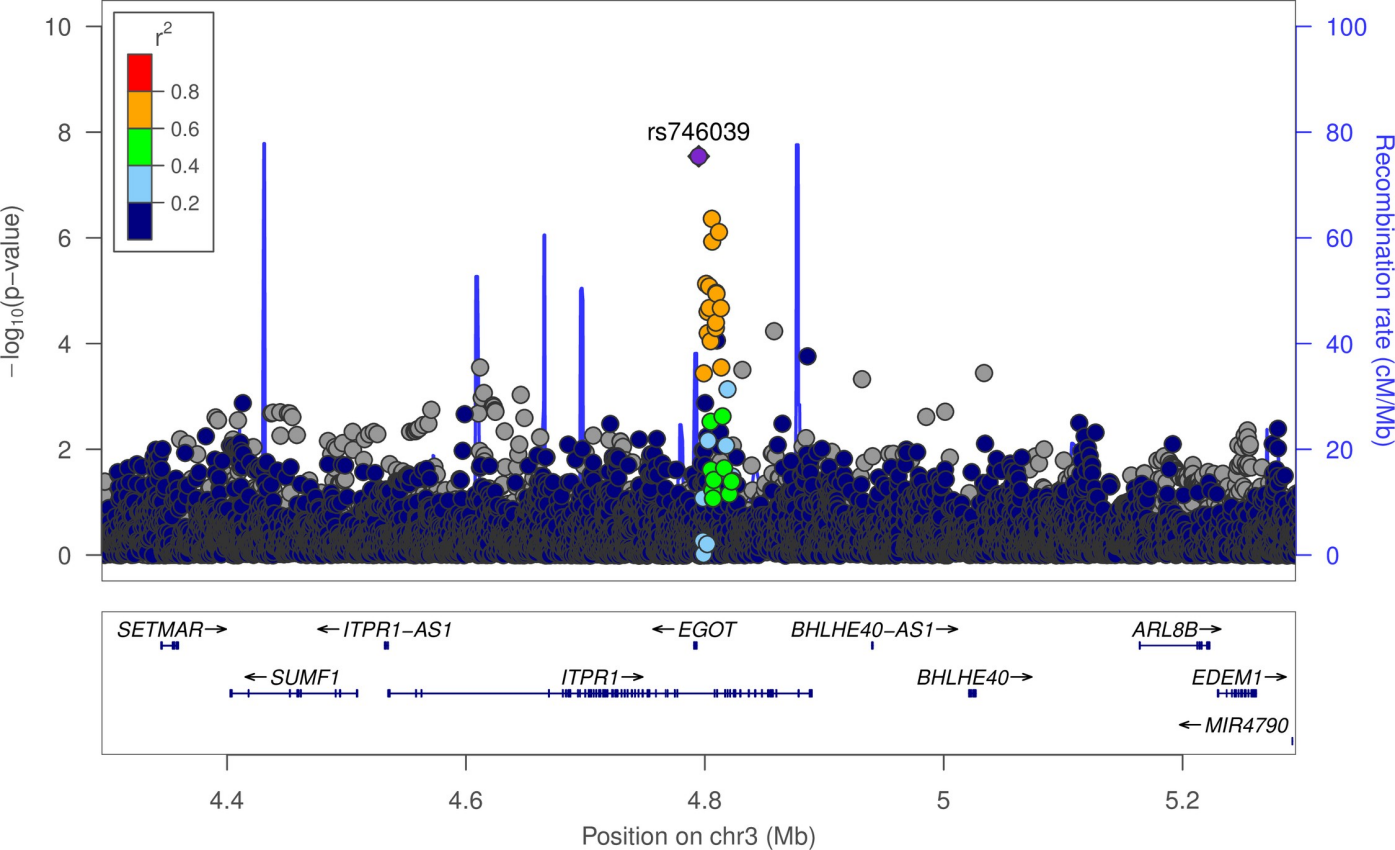
Regional plot of the *ITPR1* locus associated with fetal weight. Data span 500 kb centered at the index SNP (rs746039). The x-axis denotes genomic position and the y axis denotes the log10 *P*-value, and recombination rate (cM/Mb). The purple circle point represents the index SNP. The color of each data point indicates its linkage disequilibrium value (r^2^) with the index SNP based on HapMap2.

*Trans*-ethnic GWAS meta-analysis found genome-wide significant association of maternal rs746039 [G] (intronic in *ITPR1*) with decreased fetal weight at end of second trimester (27 weeks and 6 days gestation) (β = -0.27 s.d., *P* = 2.86x10^-8^) (Fig 1, S1 Fig). The associations remained after additional adjustment for maternal age (β = -0.26 s.d., *P* = 2.33x10^-8^).

The effect direction of the allele was consistent across the four ethnic groups. Further examination of fetal biometry measures showed that rs746039 was associated with head circumference at end of second trimester (β = -0.26 s.d., *P* = 4.85x10^-8^), but not with long bone (humerus and femur) lengths or abdominal circumference. Further *trans*-ethnic meta-analysis using a method that takes into account heterogeneity in allelic effects by ancestry [25] validated the evidence of association of rs746039 with fetal weight (log_10_Bayes factor (BF) = 6.2) and head circumference (log_10_BF = 6.1) (Table 1, S3 Table).

**Table 1:** Meta-analysis results for associations of rs746039 (*ITPR1*) with fetal biometry measures at 27 weeks and 6 days gestation.

Fetal growth measure z-score at 27 weeks gestation	Beta*	s.e.	*P*	i^2^	*P* (Q statistic)	n studies	N	Effect	logBF
Fetal weight	-0.27	0.05	2.86E-08	0.21	0.28	4	1849	----	6.2
Head circumference	-0.26	0.05	4.85E-08	0	0.43	4	1849	----	6.1
Abdominal circumference	-0.22	0.05	5.90E-06	0.57	0.07	4	1849	+---	3.1
Femur length	-0.23	0.05	4.09E-06	0	0.96	4	1849	+---	3.7
Humerus length	-0.21	0.05	2.15E-05	0	0.63	4	1849	----	3.1

*Change in fetal growth measure z-score per each G allele of rs746039 (effect allele); s.e.: standard error; logBF: log10 Bayes Factor; Effect: direction of effect in Whites, Blacks, Hispanics, and East Asians, respectively

**Fig 2:**
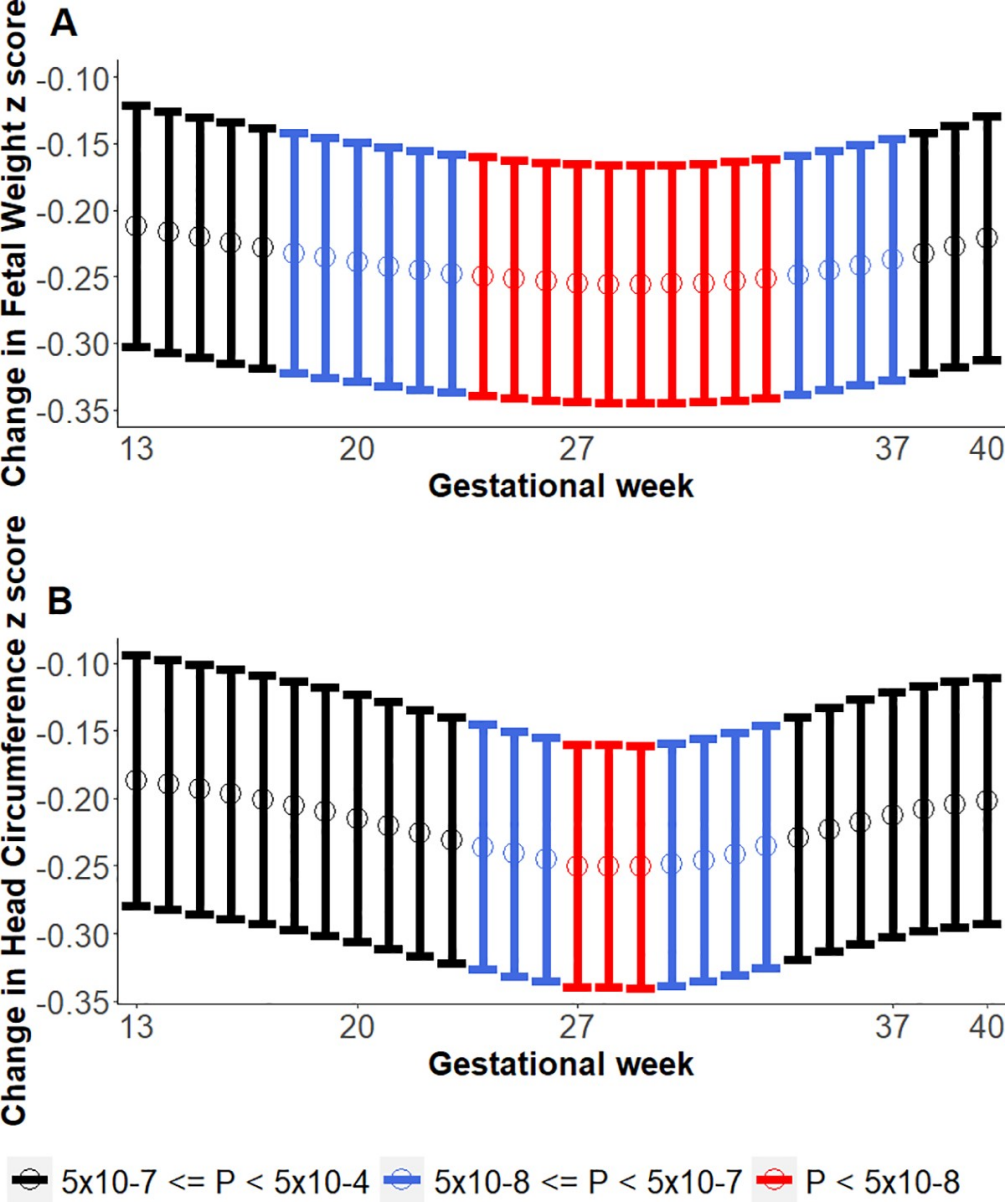
Associations of rs746039 (*ITPR1*) with fetal weight and head circumference across 13–40 weeks gestation. Y-axis shows change in fetal weight z-score per allele. Lower and upper bounds of 95% Confidence Intervals shown via the vertical lines along the mean points. Colors of vertical lines denote where genome-wide association *P*-values: red (*P* < 5x10^-8^), blue (5x10^-8^ ≤ *P* < 5x10^-7^), and black (5x10^-7^ ≤ *P* <5x10^-4^). A. Fetal weight. B. Head circumference.

To identify the gestation time window in which maternal rs746039 influenced fetal weight and head circumference across gestation, we evaluated associations between the SNP and weekly fetal weight at 13 to 40 gestational weeks. We found that rs746039 was associated with fetal weight (*P* < 5 x 10^−8^) at each week between 24 and 33 gestational weeks and with head circumference between 27 and 29 weeks (Fig 2, S4 Table).

### Replication and differentiating maternal and fetal genetic effects

Using an independent set of mother-baby dyads (n = 55 Whites, 57 Blacks and 72 Hispanics), conditional association analyses found that maternal but not fetal rs746039 G allele was significantly associated with reduced head circumference at end of second trimester (*P* = 0.04). Because the contribution of rs746039 was not nominally significant in the discovery trans-ethnic GWAS meta-analysis (*P* = 0.17), we tested the reproducibility of the meta-analysis results by excluding Blacks and confirmed that maternal but not fetal rs746039 G allele was significantly associated with reduced fetal weight (*P* = 0.02) and head circumference at end of second trimester (*P* = 0.004) (Table 2). Meta-analyses of conditional association analyses and adjusted estimates of maternal and fetal effects based on linear approximation of structural equation modelling, namely, weighted linear model (WLM) [19] showed that there was a significant difference between the independent effects of the maternal and fetal rs746039 G allele on birthweight (maternal β = -0.34, *P* = 2.34x10^-4^; fetal β = 0.15, *P* = 0.29; *P* = 2.91x10^-14^ for *F*-test for the difference between maternal and fetal effect) (S5 Table).

**Table 2:** Effects of maternal and fetal rs746039 (G) on fetal weight and head circumference at 27 weeks and 6 days gestation.

		Fetal weight *z*-score (27 weeks and 6 days)				Head circumference *z*-score (27 weeks and 6 days)			
Model	Genotype tested	Beta	s.e.	*P*	Effect	Beta	s.e.	*P*	Effect
Model 1a	Fetal	0.09	0.16	0.55	++-	0.09	0.16	0.57	++-
Model 1b	Maternal	-0.13	0.15	0.40	-+-	-0.27	0.16	0.09	-+-
Model 2a	Fetal	-0.06	0.22	0.80	++-	-0.08	0.23	0.74	++-
	Maternal	-0.18	0.17	0.27	-+-	-0.35	0.17	**0.04**	-+-
Model 2b	Fetal	-0.18	0.28	0.51	+?-	-0.13	0.28	0.64	+?-
	Maternal	-0.45	0.20	**0.02**	-?-	-0.55	0.19	**0.004**	-?-

Model 1a: fetal genotype + fetal sex. Model 1b: maternal genotype + fetal sex. Model 2: fetal genotype + maternal genotype + fetal sex (2a includes Whites, Blacks and Hispanics, 2b includes Whites and Hispanics). Effect: direction of effect based on meta-analysis with GWAMA (Whites, Blacks, Hispanics, respectively)

Through look-up of summary statistics for the most recent maternal GWAS of birthweight [19], we observed consistent direction of association. In addition, using the WLM-adjusted estimates of the independent effects of the maternal and fetal genome, Warrington *et al* [19] have found that the maternal but not fetal rs746039 G allele had birthweight-lowering effect, although the associations were not statistically significant [19].

### Fine mapping the *ITPR1* locus associations

**Fig 3:**
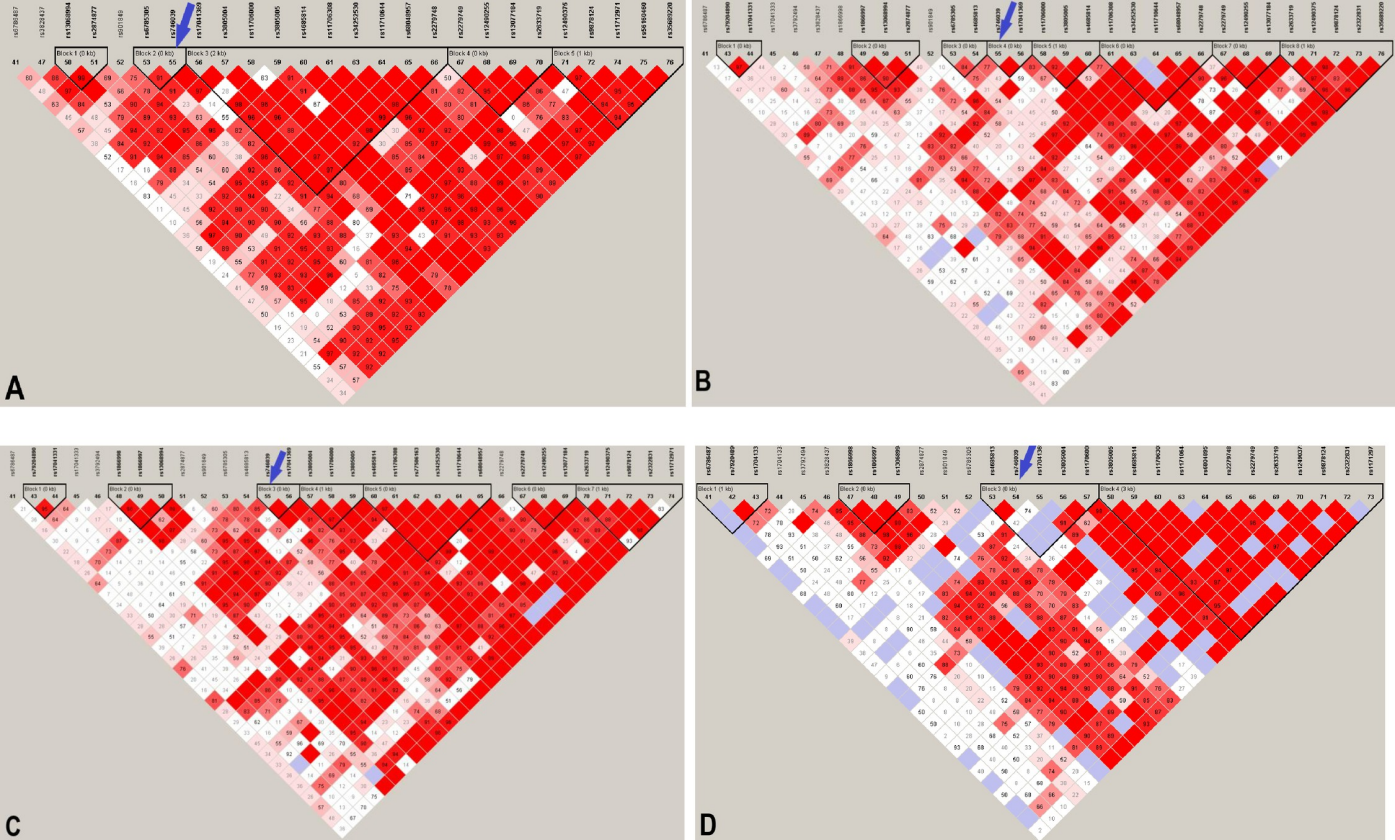
Haplotype block structure of the *ITPR1* locus associated with fetal weight among. Numbers in parenthesis denote the size, in base pair units, of the haplotype block that harbors rs746039 (marked by a blue arrow). A. White (234 bp), B. Black (69 bp), C. Hispanic (872 bp), D. East Asian (872 bp) women.

The frequencies of rs746039 G allele among the four ancestries were 20.8% in Whites, 16.3% in Hispanics, 12.5% in Blacks, and 1.6% in East Asians. The size of the haplotype block that harbors rs746039 was narrower in Blacks (69 bp) compared to Hispanics (872 bp), East Asians (872 bp), and Whites (234 bp) (Fig 3).

We leveraged this narrow haplotype structure of Blacks to further localize variants in *ITPR1* potentially causally associated with fetal weight and head circumference by performing fine mapping using the 99% credible set (CS) of the *trans*-ethnic meta-analysis with and without Blacks. The 99% CS of the four-ancestry meta-analysis of fetal weight represented six SNPs spanning 12.6 kb but was not narrower than the 99% CS from the three-ancestry meta-analysis. On the other hand, the 99% CS of the four-ancestry meta-analysis of head circumference represented four SNPs spanning 7.1 kb and was narrowed down by more than half compared to the three-ancestry 99% CS that spanned 14.6 kb (S6 Table).

### Functional annotation

Functional annotation in the HaploReg [26] database revealed that all 99% CS SNPs showed enhancer activities of the histone modification signatures H3K27ac, H3K4me1, and H3K4me3 in tissues including blood, pancreas and placenta (S6 Table). The rs746039 SNP overlapped with the binding site of the CTCF transcription factor that regulates the insulin-like growth factor 2 (*IGF2*) gene [27, 28]. Based on the mQTL database of methylation quantitative trait loci (meQTL) at serial time points across the life course [29], rs746039 was *cis*-meQTL with the CpG methylation marker cg05795849 (*P* = 1.57x10^-9^), an epigenetic signature of childhood abuse [30]. By querying the Genotype-Tissue Expression (GTEx) database [31], we found that *ITPR1* is highly expressed in the brain and uterine tissues (endometrium, fallopian tube, and ovary).

### Relationship between *ITPR1* gene expression and placental epigenetic age acceleration

**Fig 4:**
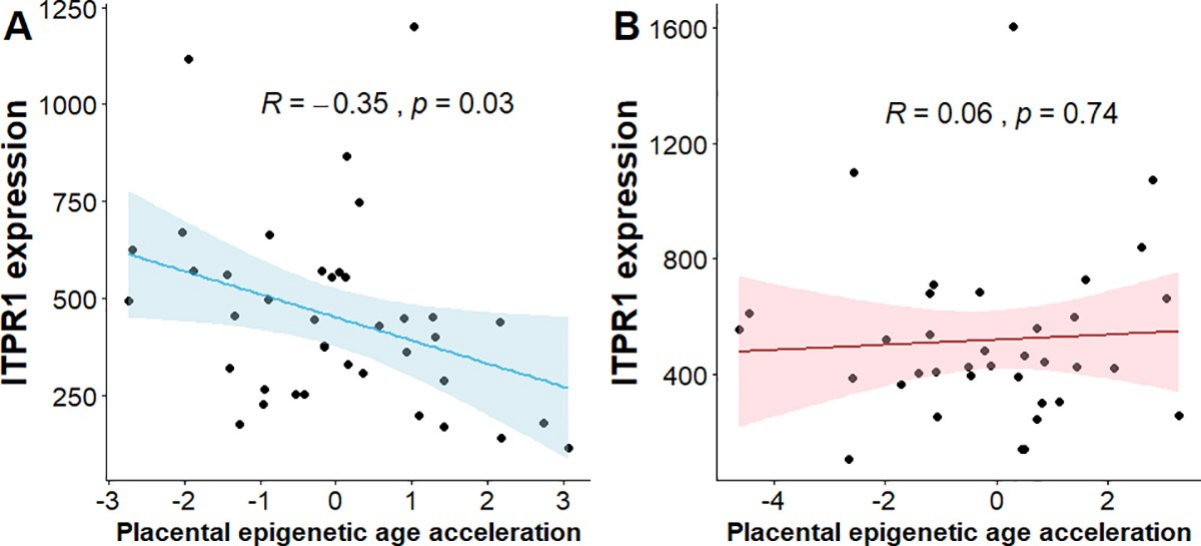
Correlations between *ITPR1* gene expression and epigenetic age acceleration in placenta. A. Male fetuses. B. Female fetuses. Lower and upper bounds of 95% Confidence Intervals shown via the corresponding colored bands around the mean lines.

Previous knockouts of *itpr1* and *itpr3* genes in mice found fetal growth retardation [32, 33]. A follow-up study found that the *IP3* genes were expressed in embryos and placenta very early during placentation [33], and the growth retardation may result from impairment of vascularization of the placenta and other extra-embryonic tissues [33]. Decreased expression of *itpr1* has also been found in aged mice skeletal muscle [34]. Therefore, we hypothesized that decreased expression of *ITPR1* in placenta may lead to accelerated aging of the tissue, an early tissue senescence that increases the risk of adverse fetal outcomes including fetal growth restriction and low birthweight [23, 24]. Placental epigenetic age acceleration was determined using a published aging “clock” estimation method [35]. We have previously shown that the methylation age of the placenta is significantly correlated with gestational age of the placenta [36]. Given the sex-biased effects of placental epigenetic age acceleration on fetal growth [36], we tested the correlation between *ITPR1* gene expression and placental epigenetic age acceleration separately in male and female fetuses. We found a significant inverse correlation between *ITPR1* gene expression and placental epigenetic age acceleration among male fetuses (r = -0.4, *P* = 0.01) but not among female fetuses (r = 0.05, *P* = 0.79) (Fig 4).

### Evaluation of published birthweight loci

**Fig 5:**
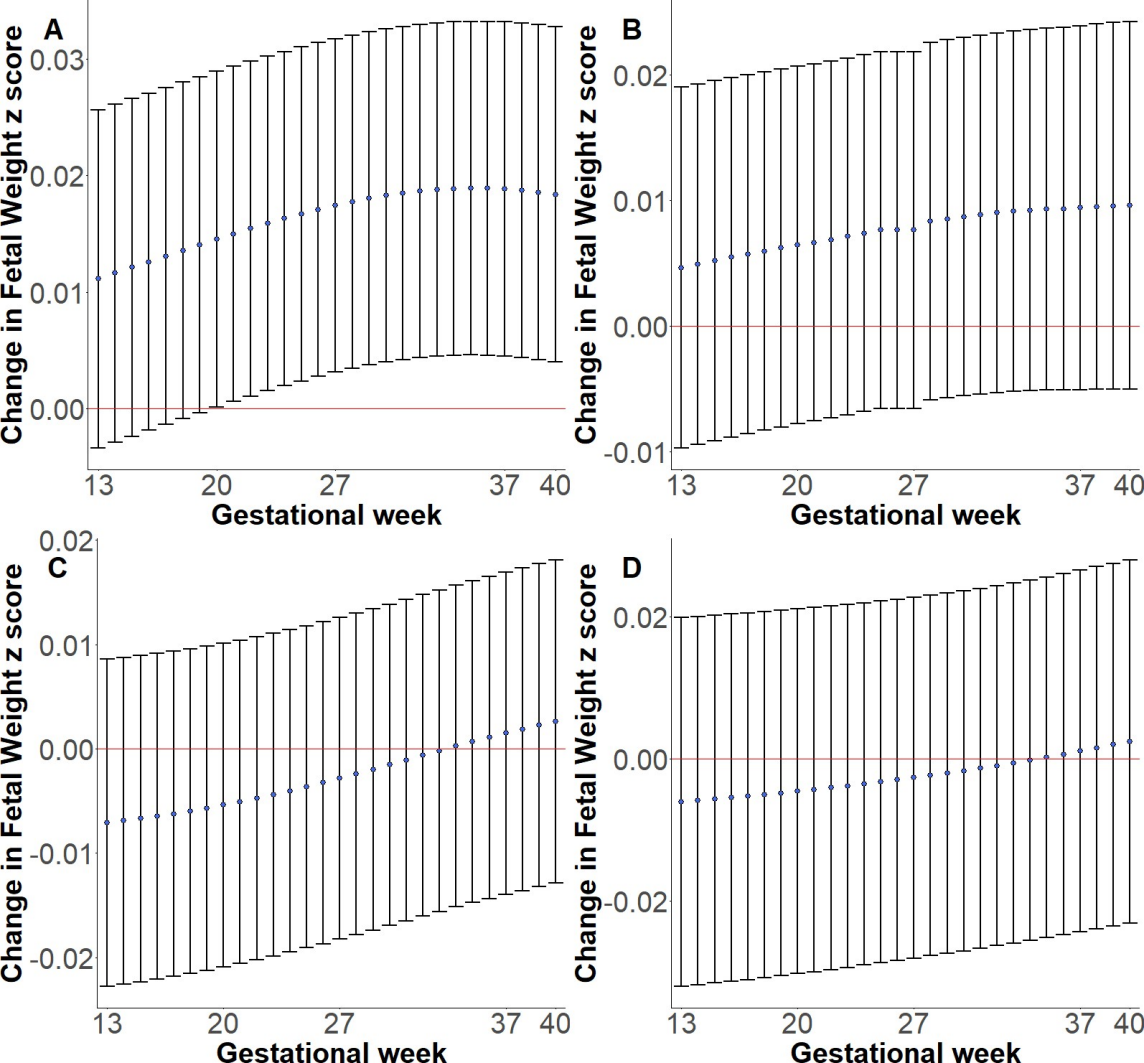
Associations between genetic risk score of birthweight-increasing maternal alleles and fetal weight across 13–40 weeks gestation. A. White, B. Black, C. Hispanic, D. East Asian. Lower and upper bounds of 95% Confidence Intervals shown via the vertical lines along the mean points.

We evaluated whether 32 SNPs found to be associated with birthweight through the maternal genome in a recent GWAS of birthweight in European ancestry populations [19] were associated with fetal weight at ends of first, second, and third trimester during gestation. A total of 31 SNPs was available in at least one ethnic group in our data. We observed that rs3918226 (*NOS3*) at end of first trimester, rs10830963 (*MTNR1B*) at ends of second and third trimesters, and rs6440006 (*ZBTB38*) and rs4579095 (*NBLA00301*) at end of third trimester were associated with fetal weight at *P* < 0.05. However, only rs10830963 (*MTNR1B*) remained significantly associated with fetal weight at end of third trimester (*P* = 9.85x10^-4^) after Bonferroni correction (*P*< 0.05/31 = 1.61x10^-3^) (S7 Table). Next, we tested the associations of a genetic risk score (GRS) formed by summing the birthweight-increasing allele dosages of the maternal SNPs with fetal weight across 13–40 weeks gestation in each ethnic group. Warrington *et al* identified a total of 105 maternal SNPs associated with birthweight at *P* < 5x10^-8^, of which 32 SNPs showed associations only through the maternal genome [19]. A total of 101 SNPs were available in at least one ethnic group and 88 SNPs were shared across the four ethnic groups in our dataset. We generated GRSs using the 88 maternal SNPs shared across the four race/ethnic groups (GRS_88_). To assess whether SNPs that may have an effect on birthweight through fetal as well as maternal genome bias the association of GRS with fetal weight, we generated two additional GRSs: (i) using 78 maternal SNPs that remained after removing SNPs resolved to have effect on birthweight through the fetal genome [19] (GRS_78_), and (ii) using the 31 SNPs annotated to have effect on birthweight only through the maternal genome (GRS_31_). Maternal birthweight GRS_88_ was associated with fetal weight in Whites across 20–40 weeks gestation (*P* < 0.05), but not in other race/ethnic groups (Fig 5, S8 Table). The association was strengthened and the effect of GRS on fetal weight was observed at earlier gestation times (16–40 weeks) when GRS was generated after SNPs with fetal effect were removed (GRS_78_) but was attenuated when a much smaller maternal-only SNPs were used (GRS_31_) (S2 Fig).

## Discussion

In this *trans*-ethnic GWAS meta-analysis, we discovered and replicated a novel association of an *ITPR1* gene locus (rs746039) in the maternal genome with fetal weight between 24–33 weeks of gestation. Through further validation of the locus in other fetal biometrics (head circumference, abdominal circumference, and femur and humerus lengths), we found that the locus was primarily associated with the fetal head circumference. *Trans*-ethnic meta-analysis that included African ancestry samples narrowed down the credible set of SNPs associated with decreased fetal head circumference as compared to one without African ancestry samples. The credible set of SNPs were found to be functional with potential regulatory effects. Lastly, our study revealed that maternal GRS of birthweight was associated with fetal weight among Whites beginning at mid-gestation.

The *ITPR1* gene, encodes the intracellular receptor IP3R1 that induces calcium release from intracellular membranes such as the endoplasmic reticulum upon stimulation by inositol 1,4,5-triphosphate [37]. IP3R1 is abundantly expressed in cerebellum followed by cerebral cortex and striatum [37]. Mutations in *ITPR1* have been linked with cerebellar atrophy on brain imaging [38], smaller head circumference [39], and a spectrum of cerebellar disorders [40]. Studies in mice also showed that homozygous *itpr1* null mice had 50% smaller weight and 40% smaller brain size compared to the wildtype mice [41]. No previous study has reported a relationship between *ITPR1* variants and fetal growth in humans. However, a GWAS of birthweight using offspring genotypes in Europeans has found an association of *ITPR2* (rs2306547), another member of the IP3R family of genes, with birthweight [42]. A SNP intronic in *ITPR2* (chr12:26958660, hg19) has been reported to be *cis*-eQTL with *ITPR2* in human placenta [43]. A recent study found that rs12812999, a SNP in strong linkage disequilibrium (LD) (r^2^ = 0.94) with the birthweight GWAS lead SNP rs2306547, influences *ITPR2* transcript levels in placenta [44].

Our conditional analysis of maternal and fetal genotype pairs in relation to fetal weight suggested that the effect of the *ITPR1* locus on fetal weight was likely due to the influence of the maternal genotype on intra-uterine factors. We found that decreased expression of *ITPR1* was correlated with accelerated epigenetic aging of the placenta, recapitulating previous studies that found decreased expression of *ITPR1* in skeletal muscle of aged mice [44], and association of placental epigenetic age acceleration with fetal growth [36]. A more direct way to assess the mechanism is first to identify the intrauterine factor influenced by rs746039, and then examine the relationship between the putative intrauterine factor and *ITPR1* expression. This is beyond the scope of our work. More detailed work in well characterized large datasets and experiments in model organisms are needed to determine the causal pathway and to translate the finding into clinical practice.

IP3R1 plays a key role in calcium (Ca^2+^) homeostasis in the endoplasmic reticulum [37], and dysregulation of endoplasmic reticulum-mitochondria Ca^2+^ cross-talk has been linked with aging in other tissues [45]. Previous GWAS found significant association of rs17041333 (1.9 kb from our *ITPR1* SNP) with urine levels of 3-hydroxypropyl mercapturic acid, a metabolite of toxicants in tobacco smoke [46]; association of rs6762644 (52.5 kb from our lead SNP) with breast cancer [47]; and suggestive association of our lead SNP with E-Selectin levels [48]. A large-scale meta-analysis of 19 cohorts involving more than 9000 mother-offspring pairs found significant association between maternal BMI at the start of pregnancy and offspring cord blood DNA methylation at cg25185429 (55.5 kb from our *ITPR1* SNP) [49]. Given the link between these phenotypes and senescence-related pathways such as oxidative stress and inflammation signaling, future functional studies may shed light into the *in-utero* link between maternal *ITPR1* variation and fetal growth.

Fetal growth restriction in early second trimester has been found to be a strong risk factor for adverse perinatal outcomes [50]. Smaller head circumference at second trimester has been associated with several neurological and chromosomal abnormalities [51]. Our finding for association between the *ITPR1* locus with fetal weight from late second trimester to early third trimester suggests that the effect of the locus may be gestation time-specific. It is possible that the locus influences intra-uterine factors during late second trimester and early third trimester, a time period when the growing fetus’s demand for oxygen and nutrients is high [13] and the placenta undergoes dramatic transitions to meet these fetal demands [52]. Consistent with these gestational age-dependent findings, we and others have previously found that the effects of fetal genetic variants on fetal growth may be gestation time-specific [53], and the influence of additive genetics on fetal weight increases with gestational age [11–13].

A unique strength of our study was presence of a multi-race/ethnic cohort that facilitated discovery of a novel locus and fine-mapping of the credible set of SNPs potentially causally associated with fetal growth. We had high-quality longitudinal measurement of fetal biometry using a standardized ultrasonology protocol with established quality control after intensive training and credentialing of sonographers [54]. However, future larger studies are needed to discover variants with low frequency and ancestry-specific effects. Our finding for associations of maternal birthweight GRS derived from GWAS involving European ancestry populations with fetal weight from mid-gestation onwards among Whites is novel. However, absence of similar associations in other ethnic groups indicates that GWAS of fetal growth are needed in more diverse populations for more accurate prediction of fetal growth across populations. Although the present study is thus far the largest GWAS on repeated fetal growth measures of diverse race/ethnicity groups, it is also possible that the small sample size of our study in each race/ethnic group, may have limited the power of the study to detect associations with small effect sizes. Another limitation of our study is that the placental samples were obtained from deliveries at or near term-pregnancy (mean ± s.d. gestational age at delivery = 39.5±1.1 weeks). Given the dynamic transcriptomic and DNA methylation changes in the placenta across gestation [55], the extent to which our findings of correlation between placental aging and *ITPR1* gene expression at term translates to earlier gestation times is not clear. Future studies of placental samples collected at different periods of gestation are needed to evaluate this. We estimated fetal weight using the Hadlock formula which was developed in 1985 using ultrasound measures of head circumference, abdominal circumference, and femur length of Caucasian women [56]. The Hadlock formula is accepted in clinical practice and research. However, an ethnic-specific formula may be needed to calculate fetal weight, but this was beyond the scope of the NICHD Fetal Growth Studies because there were only a few neonates that had an ultrasound within 1 week of delivery, and most women delivered at term pregnancy.

In conclusion, this first GWAS of longitudinal fetal growth among multi-race/ethnic cohort populations discovered a novel association of maternal *ITPR1* gene locus with fetal weight. The effect of the *ITPR1* variant on fetal weight may be modulated through intrauterine factors that influence placental function. These findings shed new light on the genetic regulation of gestation time-specific fetal growth. Future research aimed at identifying the intrauterine mechanisms that modulate the effect of the genetic variants on fetal growth may advance the translation of this discovery into clinical practice.

## Materials and methods

### Ethics statement

The study was approved by the institutional review boards of NICHD and each of the participating clinic sites. Written informed consent was obtained from all study participants.

### Data set

Self-identified Hispanic, non-Hispanic Black, non-Hispanic White, and Asian/Pacific Islander pregnant women from the NICHD Fetal Growth Studies–Singletons were included in our analysis. Briefly, the NICHD Fetal Growth Studies–Singletons was a multi-ethnic prospective cohort study designed to establish standards for fetal growth in the U.S. population. A total of 2,802 pregnant women were recruited between 8–13 gestational weeks between July 2009 and January 2013 at 12 clinic sites in the U.S. Detailed recruitment and inclusion criteria of the study have been reported previously [57].

### Ultrasonography measures of fetal growth and quality control

The study implemented a standardized ultrasonography protocol with established quality control. All sonographers underwent an intensive training and evaluation period [54], and fetal growth measurements were performed using an identical equipment (Voluson E8; GE Healthcare, Milwaukee, WI) [57]. The quality control showed that ultrasound measurements between site sonographers and experts had high correlation (> 0.99) and low coefficient of variation (< 3%) [54].

After the first ultrasound to confirm gestational age, pregnant women underwent five standardized ultrasounds at *a priori* defined gestational ages. At each ultrasound visit, head circumference, biparietal diameter, abdominal circumference, humerus length, and femur length were measured. Estimated fetal weight was calculated from head circumference, abdominal circumference, and femur length using the Hadlock formula [58]. Details of the fetal growth trajectory estimation models implemented in the NICHD Fetal Growth Studies cohort have been previously described [10]. Briefly, fetal growth trajectories were created using a linear mixed model with a cubic spline mean structure and a cubic polynomial random effect. Briefly, knot points at 25^th^, 50^th^ and 75^th^ percentiles were fixed at evenly distributed gestation weeks and percentiles were estimated considering random effects and error structure, ultimately estimating the growth curves.

### Genotyping and quality control

DNA was successfully extracted from stored buffy coat specimens obtained from 582 Hispanics, 652 Blacks, 641 Whites, and 340 Asians. Maternal SNPs were genotyped using the Infinium Multiethnic Global BeadChip microarray (Illumina) that has ~1.7 million SNPs. Quality control of genome-wide SNP data was carried out within each race/ethnic group using PLINK version 1.9 [59]. We excluded participants with >5% missing SNP genotypes, high degree of relatedness (Pi hat ≥0.25), and excess heterozygosity (≥3 S.D. from the mean). We generated principal components (PCs) from multi-dimensional scaling analysis of a pruned set of uncorrelated genome-wide SNPs (i.e., r^2^ threshold of 0.2, and a sliding window of 50 SNPs by skipping 5 SNPs between consecutive windows) as implemented in the program PLINK. Based on the multi-dimensional scaling analysis plots, outliers from the distribution of the Hispanic, African, European, and East Asian clusters of the 1000 Genomes reference population were removed. We removed insertion-deletions, multi-allelic and duplicated SNPs. We also removed SNPs with >5% missing values, minor allele frequency <0.5%, and not in Hardy-Weinberg equilibrium (*P* <10^−4^). After quality control, 622 Whites with 825,185 SNPs, 637 Blacks with 1,078,089 SNPs, 568 Hispanics with 1,044,163 SNPs, and 238 Asians with 748,179 SNPs remained. The overall genotyping call rate was 99.7% in Whites, 99.6% in Blacks, 99.8% in Hispanics, and 99.3% in Asians (S9 Table).

### Imputation and quality control

SNP genotypes were imputed using the Michigan Imputation Server implementing Eagle2 for haplotype phasing, followed by Minimac2 for imputing non-typed SNPs using the 1000 Genomes Phase 3 genotype dataset [60]. We applied filters to remove insertion-deletions, SNPs with minor allele frequency <0.005 and SNPs with dosage r^2^ (the squared correlation between imputed allele dosages and true experimental genotypes [61]) <0.3. The final imputed genotype data had 10 million SNPs in Whites, ~18 million SNPs in Blacks, ~14 million SNPs in Hispanics, and ~9 million SNPs in East Asians. The median dosage r^2^ of the imputed SNPs that remained after quality filters was 0.97 in Whites, 0.94 in Blacks, 0.96 in Hispanics, and 0.96 in East Asians. A total of 580 Whites, 556 Blacks, 508 Hispanics, and 205 Asians with two or more ultrasound measures and live birth data and non-missing fetal sex were included in subsequent analyses (S9 Table).

### Ethnic-specific GWAS analysis

In each of the four race/ethnic populations, linear regression analysis was performed to test the associations of maternal SNPs with z-score transformed fetal weight at 13 weeks and 6 days, 27 weeks and 6 days and 40 weeks and 0 day. The analyses were performed under an additive genetic model that adjusted for fetal sex and the first ten PCs as implemented in MACH2QTL [61].

### *Trans*-ethnic meta-analyses

The four ethnic-specific GWAS summary statistics were combined using two approaches. First, a random-effects meta-analysis was done using GWAMA (Genome-Wide Association Meta-Analysis) that accounts for heterogeneity in allelic effects between distantly related populations [62]. Heterogeneity of the associations across the four ethnicities was tested using Cochran's Q statistic and I^2^, and the *P* value of the chi-square was used to declare significance, as reported by GWAMA[62]. Genome-wide significance was set as *P* value < 5×10^− 8^. The distribution of the test statistic was consistent with the expected distribution under the null hypothesis as shown by the quantile-quantile (QQ) plots. Genomic inflation factor (λ) ranged between 0.957 and 1.008 for ancestry-specific associations and was 0.994 for the *trans*-ethnic meta-analysis, indicating a lack of population structure (S3 Fig). Plots of principal components have been presented in S4 Fig. Next, ethnic-specific GWAS summary statistics were combined using MANTRA (Meta-Analysis of Trans-ethnic Association Studies) that accounts for heterogeneity in allelic effects due to differences in linkage disequilibrium (LD) structure by ancestry [25]. MANTRA uses estimated effect sizes, standard errors and allele frequencies of each study to assign each population to ‘cluster’ according to the Bayesian partition model and evaluate the statistical significance [25]. Evidence of association was assessed by means of Bayes factor (BF), which represents the probability under the alternative hypothesis that the SNP is associated with the outcome divided by the probability under the null hypothesis of no association. Association results with log_10_BF of 6.1 or above were considered to be genome-wide significant [63].

### Evaluation of GWAS locus in other fetal biometrics

Fetal weight is a composite of multiple fetal biometry including fat-free mass, head bone, and long skeletal bones. To understand the fetal biometrics that may be predominantly influenced by the genetic loci identified through GWAS of fetal weight, we evaluated the significantly associated locus in our primary analyses for associations with other fetal growth measures including head circumference, abdominal circumference, humerus length, and femur length at the corresponding gestation week. The analyses results were then pooled using trans-ethnic meta-analysis as implemented in GWAMA.

### Fine mapping with haplotype blocks and 99% credible sets

First, we constructed the haplotype map of the genetic region that harbors the GWAS significant locus in each race/ethnic population. The haplotype block structure ± 5kb from the GWAS SNP was determined based on haplotypes with ≥ 5% occurrence, using the 4-gamete rule as implemented in Haploview 4.0 [64]. The haplotype block sizes were compared among the race/ethnic groups. To fine-map the locus showing genome-wide significance in the GWAS meta-analysis, we constructed the 99% credible sets which represent the minimum set of variants accounting for 99% of posterior probabilities in the region [65]. SNPs in the credible sets are most likely to be causal based on the statistical evidence from the *trans*-ethnic meta-analysis. Assuming a single causal variant for each locus and that the true causal variant is either genotyped or well imputed, the probability that the 99% credible set contains the causal variant would be ∼0.99. We first calculated the posterior probabilities for each variant located ±500 kb from the top variant (variant showing the lowest *P*-value in the region), i.e. the corresponding Bayes Factor (BF) divided by the summation of BF over all variants in the region. Variants were then ranked according to their BF and the ranked variants were combined to form the credible set until their cumulative posterior probability attains or exceeds 99%. To evaluate whether the trans-ethnic meta-analysis helped narrow down the association region, we constructed two sets of 99% credible sets: one that includes three race/ethnic groups (i.e., Whites, Hispanics and East Asians) and another that included Blacks in addition to the other three populations. We chose this approach because the weaker LD and narrow haplotype structure of African genomes may facilitate fine-mapping of the locus as demonstrated by other studies [66].

### Functional annotation of credible set variants

We annotated the credible set variants for regulatory potentials against the HaploReg [26] database. More specifically, we explored evidence of transcription factor binding, DNase hypersensitivity as well as promoter and enhancer activities via histone modification signals. Active promoter is marked by signature of H3K4me3, whereas H3K27ac and H3K4me1 define regions with enhancer activity. Additionally, we conducted methylation quantitative loci (meQTL) analyses to test for SNP influences on DNA methylation at different life stages in human blood: pregnancy, childhood and adolescence. We tested for enrichment of our top-significant CpGs for being targets of meQTL associations by querying them in a large-scale genome-wide DNA methylation analysis of 1,000 mother-child pairs at serial time points across the life-course [29]. Gene expression was examined across several tissues using the Genotype-Tissue Expression (GTEx) [67] database.

### Replication and maternal-offspring pair genetic analysis

Maternal genetic contributions to offspring birthweight can be due to maternally inherited offspring risk alleles or the influence of maternal genotypes on components of the intrauterine environment [18, 21]. We attempted to distinguish whether the locus found to be associated with fetal weight was due to maternal or fetal genotype effect. We examined this in 184 mother-offspring dyads (55 White, 57 Black and 72 Hispanic) and 242 offspring (72 White, 71 Black and 99 Hispanic) from the NICHD Fetal Growth Studies. Genotyping was performed on the Illumina HumanOmni2.5 Beadchips (Illumina Inc., San Diego, CA) using stored buffy coat specimens of maternal blood samples and fetal-side placental biopsies for the offspring. We performed linear regression analysis testing additive effects of fetal and maternal genotypes on z-scores of fetal weight and head circumference, each in four models. Models 1a and 1b separately tested the effects of fetal and maternal genotypes, respectively. Model 2 included both fetal and maternal genotypes. All models included fetal sex as a covariate. WLM-adjusted estimates of the independent effects of maternal and fetal effects were calculated from the unadjusted estimates of the fetal (n = 242) and maternal (n = 1849) meta-analyses results as implemented in Warrington *et al* [19]. Inverse variance-weighted meta-analysis was used to combine the estimated from the WLM-adjusted analyses and conditional analyses of mother-offspring dyads, and the difference between the fetal and maternal independent effects was estimated using an *F*-test.

### Correlation between gene expression and placental epigenetic age acceleration

Placental samples were obtained at delivery from 312 women as part of the *Eunice Kennedy Shriver* National Institute of Child Health and Human Development (NICHD) Fetal Growth Studies—Singletons. The mean ± s.d. gestational age at birth during which placental samples were collected was 39.5±1.1 weeks. DNA from placental biopsies was extracted and assayed using Illumina’s Infinium Human Methylation450 Beadchip (Illumina Inc., San Diego, CA). Processing of the placental biopsies, and DNA methylation data quality control has been described previously [68]. RNA was extracted from 80 placentas using TRIZOL reagent (Invitrogen, MA), and sequenced using the Illumina HiSeq2000 system with 100 bp paired-end reads. Following base-calling and mapping of reads, transcript abundance was quantified using Salmon [69]. Placental DNA methylation age was predicted using 62 CpGs that have previously been found to predict placental DNA methylation age with high accuracy [35]. Placental DNA methylation age acceleration was defined as the residual resulting from regressing DNA methylation age of the placenta on gestational age at delivery. The correlation between *ITPR1* gene expression and placental age acceleration was tested using Pearson correlation, separately in male (n = 39) and female (n = 36) fetuses.

### Evaluation of published birthweight SNPs

We evaluated whether maternal SNPs at 105 loci that were previously implicated in offspring birthweight in GWAS [19] were associated with *in-utero* growth in our study. Out of the 105 SNPs, 101 SNPs were found in two or more ethnicities and 88 SNPs were found across the four ethnic groups (S10 Table). For each ethnic group, we tested the associations between the SNPs and fetal weight at end of first, second, and third trimester, with adjustment for the first five genotype principal components and fetal sex. The outputs of the race/ethnic-specific analyses were combined using trans-ethnic meta-analysis implemented in GWAMA. Meta-analysis *P* < 0.05 with the same effect directions as the published birthweight GWAS was considered to be significant.

### Genetic risk score (GRS) construction and analysis

We constructed an unweighted GRS for each woman by summing the dosage of the birthweight-increasing allele [19] for each of the 88 SNPs found across the four ethnic groups in our study. We preferred using an unweighted GRS because it has been shown to be more robust to errors arising from differences in effect size and population structure, particularly among our ancestrally diverse race/ethnic groups as recommended previously [70]. Linear regression analysis was performed to test the associations between GRS and fetal weight across 13 to 40 weeks gestation in each race/ethnic group after adjusting for fetal sex and the first five genetic principal components. All analyses, unless specified otherwise, were implemented using the software package R version 3.1.2 (R Development Core Team).

## Supporting information

S1 Table Characteristics of the study participants. (XLSX)

S2 Table Pearson correlations between maternal characteristics and fetal growth measures. (XLSX)

S3 Table Associations of rs746039 (*ITPR1*) with fetal growth measures at 27 weeks and 6 days gestation. (XLSX)

S4 Table *Trans*-ethnic meta-analysis of associations between rs746039 (G) and fetal weight and head circumference across 13–40 weeks' gestation. (XLSX)

S5 Table Adjusted independent effects of maternal and fetal rs746039 (G) on fetal weight at 27 weeks and 6 days gestation. (XLSX)

S6 Table 99% credible set single nucleotide polymorphism annotations. (XLSX)

S7 Table Genetic loci associated with birthweight through the maternal genome and evaluation of their association with fetal weight across 13–40 weeks gestation. (XLSX)

S8 Table Association between birthweight-increasing genetic risk score and fetal weight across 13–40 weeks gestation. (XLSX)

S9 Table Quality control of single nucleotide polymorphisms and samples. (XLSX)

S10 Table Allele frequency and genotyping characteristics of 104 maternal SNPs known to be associated with birthweight. (XLSX)

S1 Fig Manhattan plot of genome-wide *trans*-ethnic meta-analysis of fetal weight at end of second trimester. (DOCX)

S2 Fig Genetic risk score of birthweight-increasing maternal alleles and fetal weight across 13–40 weeks gestation. In S2A, 31 SNPs associated with birthweight only through the maternal genome are included. In S2B, 77 SNPs associated with birthweight through maternal or fetal genome are included. A. White, B. Black, C. Hispanic, D. East Asian. Lower and upper bounds of 95% Confidence Intervals shown via the vertical lines along the mean points.
(DOCX)

S3 Fig Quantile-quantile plots of p-values of genome-wide associations with fetal weight at end of second trimester. The five quantile-quantile plots represent the following: A. White, B. Black, C. Hispanic, D. East Asian, E. Meta-analysis.
(DOCX)

S4 Fig Plots of the first three principal components. (DOCX)
