# Supplementary material for: Trans-ethnic meta-analysis of genome-wide association studies identifies maternal ITPR1 as a novel locus influencing fetal growth during sensitive periods in pregnancy
Source: PLoS Genet. 2020 May 14;16(5):e1008747. doi: 10.1371/journal.pgen.1008747 (PMC7252673; doi:10.1371/journal.pgen.1008747)

**S1 Fig**. Manhattan plot of genome-wide *trans*-ethnic meta-analysis of fetal weight at end of second trimester (27 weeks and 6 days gestation)
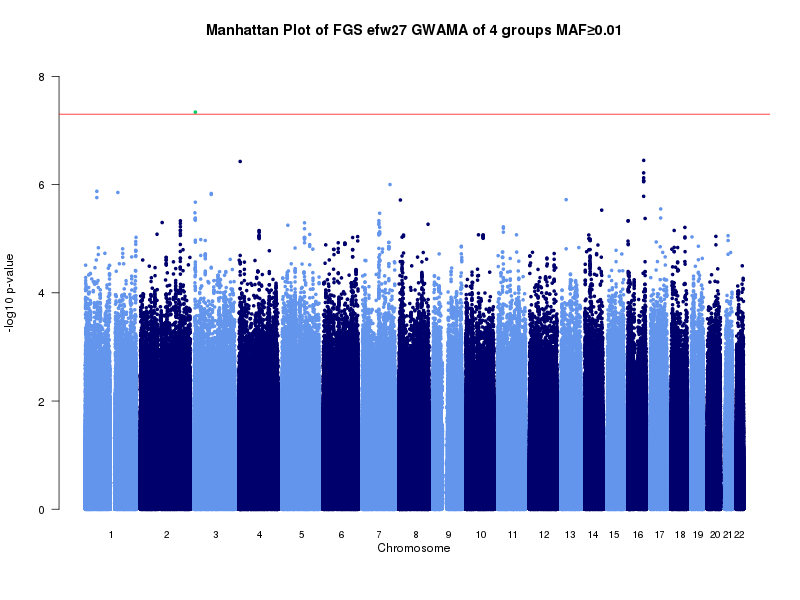

Supplement: S1 Fig — (DOCX) [file pgen.1008747.s011.docx]
