# Supplementary material for: Trans-ethnic meta-analysis of genome-wide association studies identifies maternal ITPR1 as a novel locus influencing fetal growth during sensitive periods in pregnancy
Source: PLoS Genet. 2020 May 14;16(5):e1008747. doi: 10.1371/journal.pgen.1008747 (PMC7252673; doi:10.1371/journal.pgen.1008747)
Supplement: S2 Fig — In S2A, 31 SNPs associated with birthweight only through the maternal genome are included. In S2B, 77 SNPs associated with birthweight through maternal or fetal genome are included. A. White, B. Black, C. Hispanic, D. East Asian. Lower and upper bounds of 95% Confidence Intervals shown via the vertical lines along the mean points. (DOCX) [file pgen.1008747.s012.docx]

**S2 Fig. Genetic risk score of birthweight-increasing maternal alleles and fetal weight across 13-40 weeks gestation.**

In (S2A) 31 SNPs associated with birthweight only through the maternal genome are included. In (S2B), 78 SNPs associated with birthweight through maternal or fetal genome are included. A. White, B. Black, C. Hispanic, D. East Asian. Lower and upper bounds of 95% Confidence Intervals shown via the vertical lines along the mean points.

| **S2A: maternal only GRS (GRS31)** |
| --- |
| 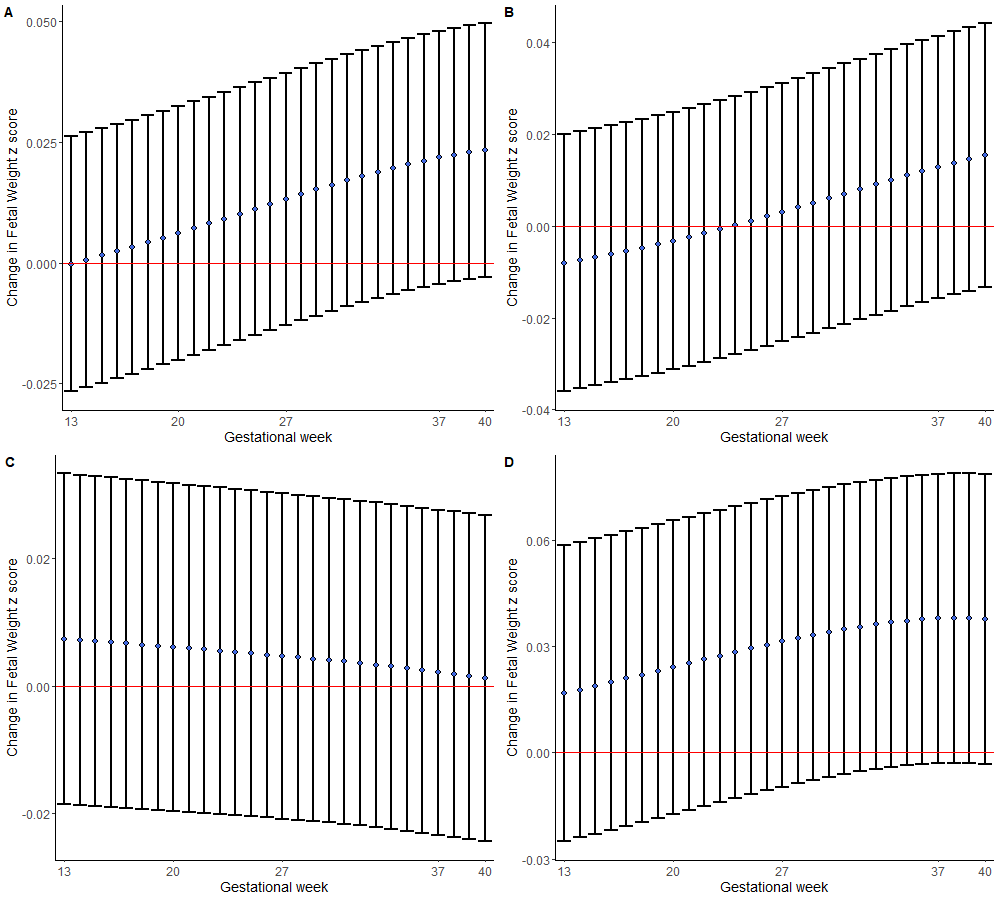 |
| **S2B: maternal GRS (GRS78)** |
| 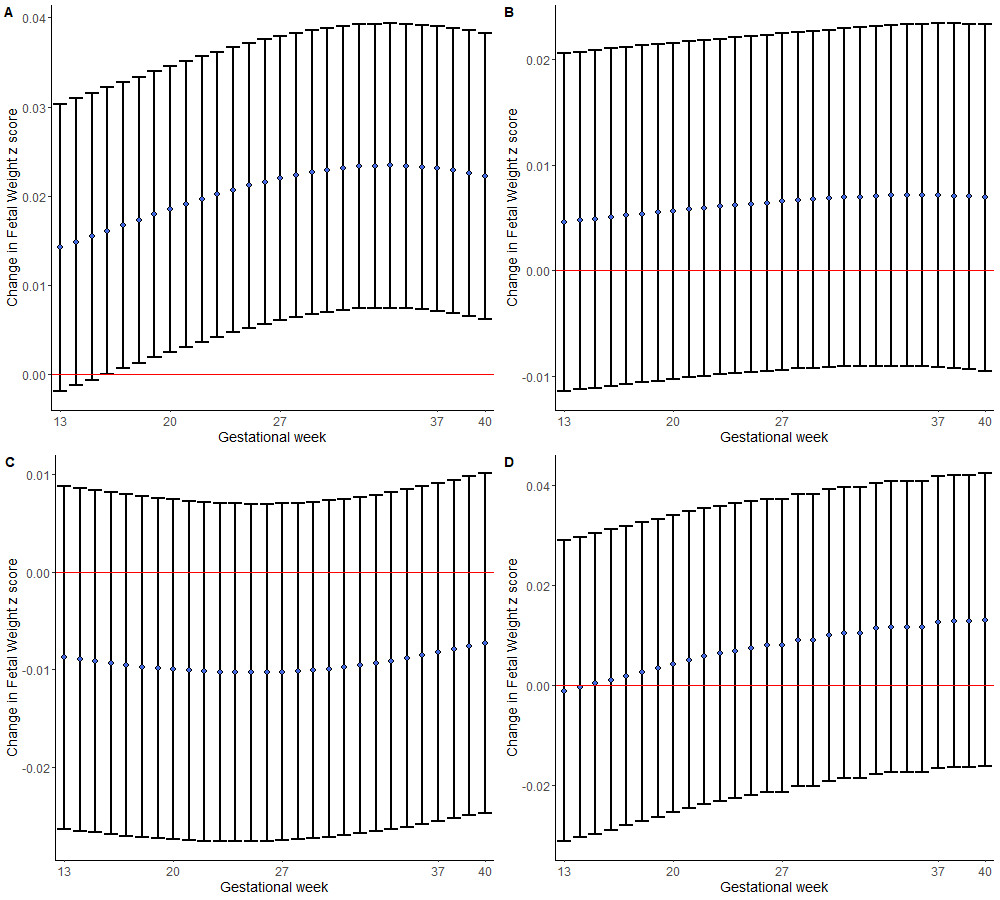 |
