# Supplementary material for: Trans-ethnic meta-analysis of genome-wide association studies identifies maternal ITPR1 as a novel locus influencing fetal growth during sensitive periods in pregnancy
Source: PLoS Genet. 2020 May 14;16(5):e1008747. doi: 10.1371/journal.pgen.1008747 (PMC7252673; doi:10.1371/journal.pgen.1008747)
Supplement: S3 Fig — The five quantile-quantile plots represent the following: A. White, B. Black, C. Hispanic, D. East Asian, E. Meta-analysis. (DOCX) [file pgen.1008747.s013.docx]

**S3 Fig**. Quantile-quantile plots of p-values of genome-wide associations with fetal weight at end of second trimester (27 weeks and 6 days gestation)

| **A. White** | **B. Black** |
| --- | --- |
| 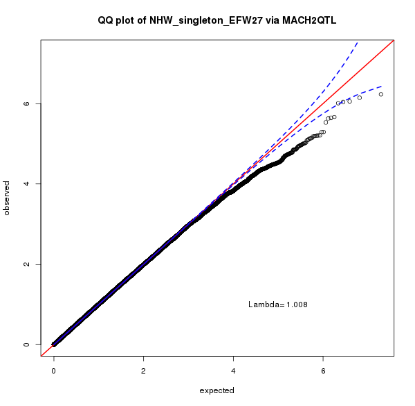 | 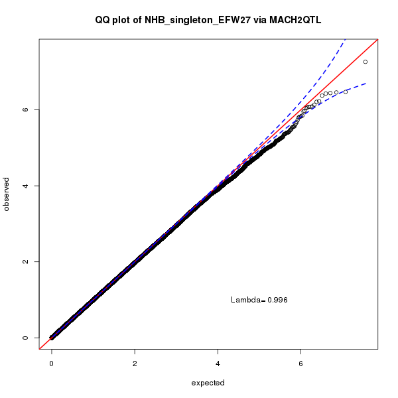 |
| **C. Hispanic** | **D. East Asian** |
| 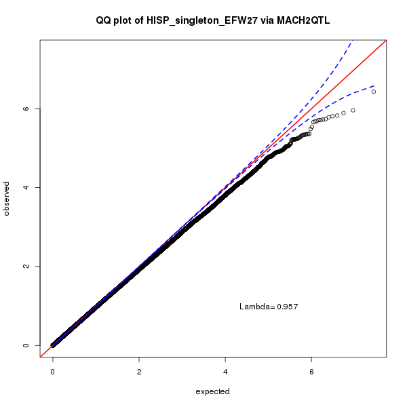 | 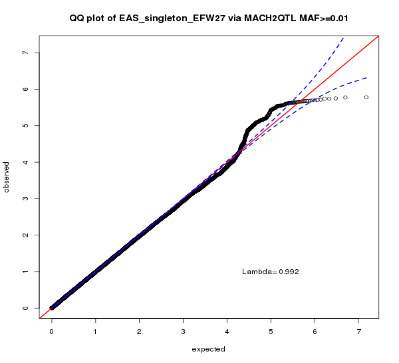 |
| **E. Meta-analysis** |  |
| 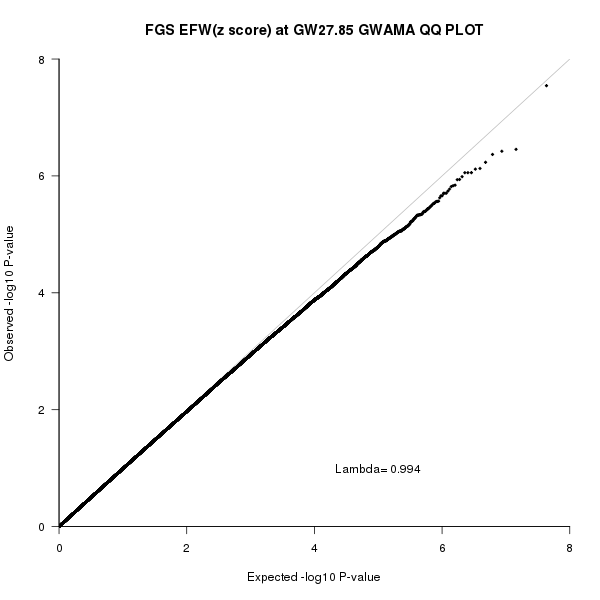 |  |
