# Supplementary figures and images for: Trans-ethnic meta-analysis of genome-wide association studies identifies maternal ITPR1 as a novel locus influencing fetal growth during sensitive periods in pregnancy
Source: PLoS Genet. 2020 May 14;16(5):e1008747. doi: 10.1371/journal.pgen.1008747 (PMC7252673; doi:10.1371/journal.pgen.1008747)

**S4 Fig.** Plots of the first three principal components.

| **White** |
| --- |
| 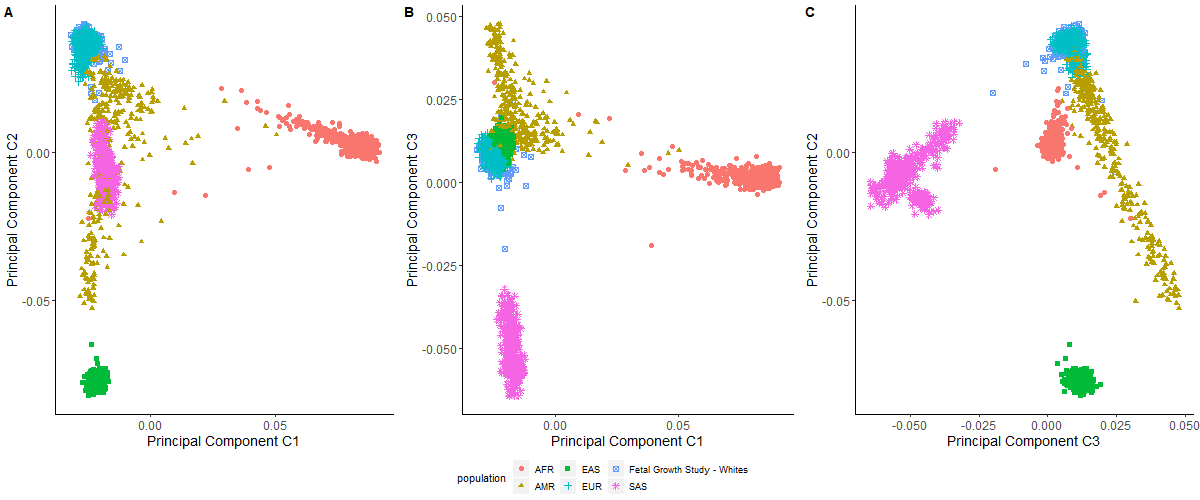 |
|  |
| **Black** |
| 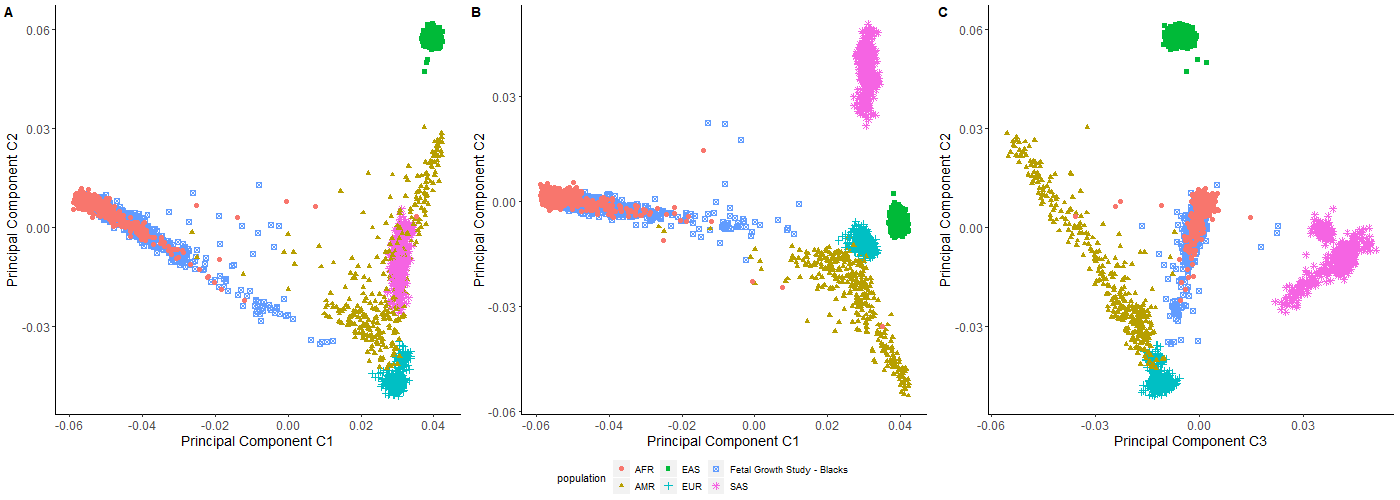 |
| **Hispanic** |
| 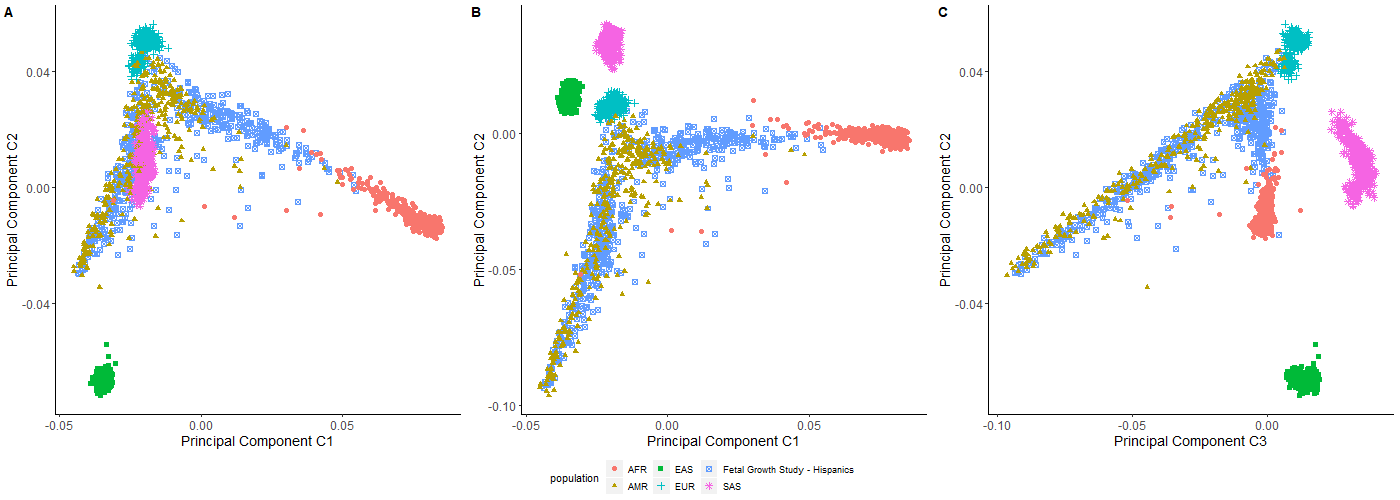 |
| **East Asian** |
| 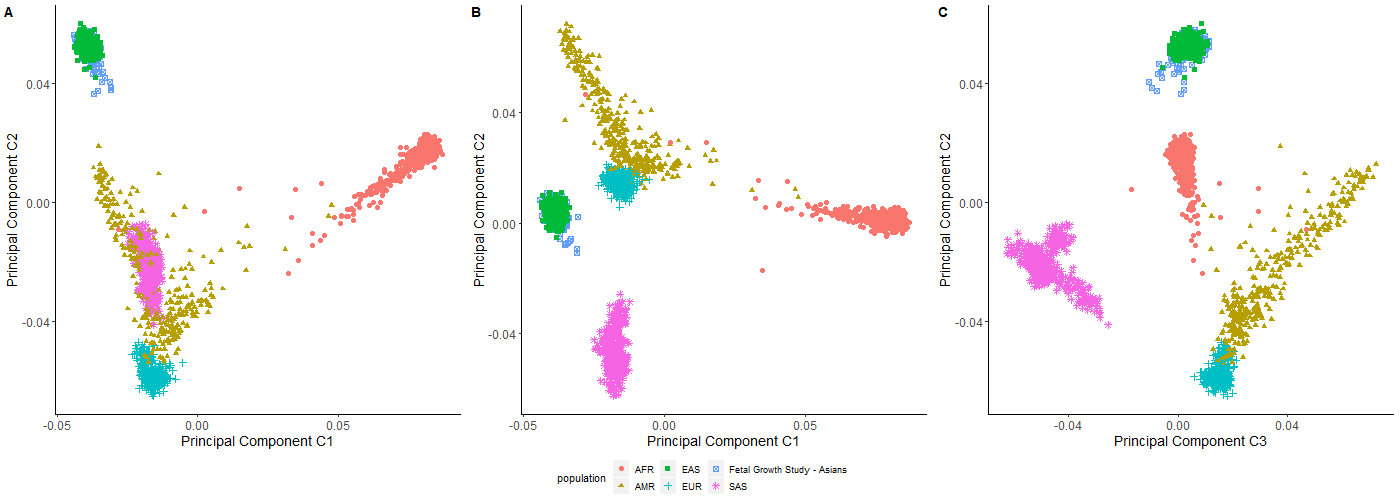 |
| 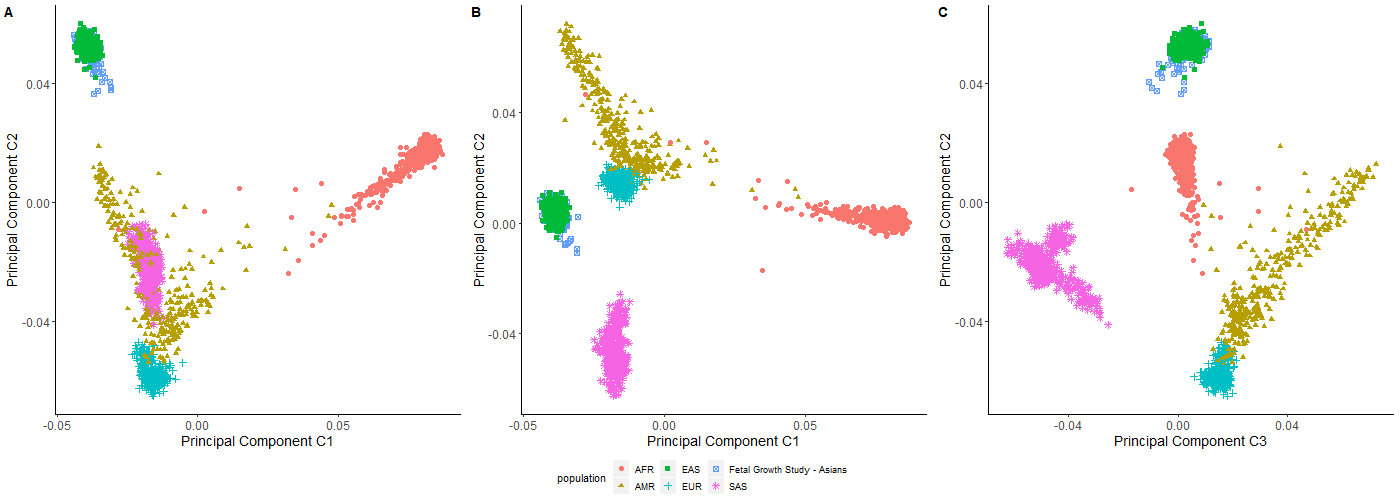 |

Supplement: S4 Fig — (DOCX) [file pgen.1008747.s014.docx]
